# PET/CT standardized uptake value and EGFR expression predicts treatment failure in nasopharyngeal carcinoma

**DOI:** 10.1186/s13014-023-02231-6

**Published:** 2023-02-22

**Authors:** Zhaodong Fei, Ting Xu, Huiling Hong, Yiying Xu, Jiawei Chen, Xiufang Qiu, Jianming Ding, Chaoxiong Huang, Li Li, Jing Liu, Chuanben Chen

**Affiliations:** Department of Radiation Oncology, Fujian Cancer Hospital, Clinical Oncology School of Fujian Medical University, Fuma Road, Fuzhou, 350014 Fujian People’s Republic of China

**Keywords:** Nasopharyngeal carcinoma, PET/CT, Standardized uptake values, EGFR

## Abstract

**Objective:**

This study inventively combines epidermal growth factor receptor (EGFR) expression of the primary lesion and standardized uptake value (SUV) of positron emission tomography and computed tomography (PET/CT) to predict the prognosis of nasopharyngeal carcinoma (NPC). This study aimed to evaluate the predictive efficacy of maximum standard uptake value (SUVmax) and EGFR for treatment failure in patients with NPC.

**Methods:**

This retrospective study reviewed the results of EGFR expression and pretreatment ^18^F-FDG PET/CT of 313 patients with NPC. Time-dependent receiver operator characteristics was used for analyzing results and selecting the optimal cutoff values. Cox regression was used to screen out multiple risk factors. Cumulative survival rate was calculated by Kaplan–Meier.

**Results:**

The selected cutoff value of SUVmax-T was 8.5. The patients were categorized into four groups according to EGFR expression and SUVmax-T. There were significant differences in the 3-year local recurrence-free survival (LRFS) (*p* = 0.0083), locoregional relapse-free survival (LRRFS) (*p* = 0.0077), distant metastasis-free survival (DMFS) (*p* = 0.013), and progression-free survival (PFS) (*p* = 0.0018) among the four groups. Patients in the EGFR-positive and SUVmax-T > 8.5 group had the worst survival, while patients in the EGFR-negative and SUVmax-T ≤ 8.5 group had the best prognosis. Subsequently, patients with only positive EGFR expression or high SUVmax-T were classified as the middle-risk group. There were also a significant difference in 3-year overall survival among the three risk groups (*p* = 0.034). SUVmax-T was associated with regional recurrence-free survival and LRRFS in multivariate analysis, whereas EGFR was an independent prognostic factor for LRRFS, DMFS, and PFS.

**Conclusion:**

The combination of SUVmax-T and EGFR expression can refine prognosis and indicate clinical therapy.

**Supplementary Information:**

The online version contains supplementary material available at 10.1186/s13014-023-02231-6.

## Introduction

Nasopharyngeal carcinoma (NPC) is a highly aggressive malignant tumor believed to arise from nasopharyngeal epithelial cells [1]. According to the International Agency for Research on Cancer, in 2020, there were approximately 133,000 new cases and 80,000 new deaths due to NPC [2]. Nevertheless, more than 70% of new cases are from East and Southeast Asia, and China has the largest number of deaths worldwide [1, 2]. Platinum-based concurrent chemoradiotherapy (CCRT) and radiotherapy (RT) are the standard treatments for patients with NPC [3]. In the era of intensity-modulated RT (IMRT), the 5-year overall survival (OS) rate could reach more than 80% [4, 5]. While the rate of recurrence and/or metastasis ranges from 20 to 30%, which is the predominant cause of treatment failure [6, 7]. Therefore, early identification of recurrence and metastasis and the subsequent development of more aggressive de novo treatment are key to improving local tumor control and survival.

Traditionally, the TNM clinical stage has been considered the most important prognostic factor for NPC based on anatomic imaging. However, the prognosis is different for patients with no difference TNM stage treated by the same treatment regimen. Hence, for conventional staging system carries on the optimization and investigating the new index can better predict clinical outcomes. Recently, ^18^F-fluorodeoxyglucose positron emission tomography and computed tomography (^18^F-FDG PET/CT) have been frequently used in pretreatment diagnostic evaluation and post-treatment monitoring because of their unique capability to image metabolically active lesions [8, 9]. PET/CT enables the quantitative assessment of biochemical, physiological, and metabolic alterations in vivo by integrating morphologic and functional imaging [10, 11]. Several investigators have confirmed the value of semiquantitative parameters of PET, such as standardized uptake value (SUV), termed metabolic tumor volume (MTV), and total lesion glycolysis (TLG), for predicting the prognosis of NPC [12–15]. Our previous study also demonstrated that the maximum standard uptake value (SUVmax) can reflect the biologic aggressiveness and metastatic potential of NPC [16].

Epidermal growth factor receptor (EGFR) overexpression is observed in more than 90% of NPC cases, and EGFR expression is associated with poor prognosis [17, 18]. Altered EGFR signaling is widely implicated in NPC cell proliferation, angiogenesis, invasion, and metastasis, which may lead to treatment resistance and poor survival outcome [19, 20]. However, no conclusive results have been drawn as to whether PET/CT parameters, especially SUVmax, combined with EGFR could better predict the prognosis of NPC.

Hence, this study inventively combined EGFR expression of the primary lesion and pretreatment SUVmax of PET/CT to predict the prognosis of NPC. The present study aimed to evaluate the predictive efficacy of SUVmax and EGFR for treatment failure in NPC patients.

## Materials and methods

### Patients

A retrospective analysis of related medical records of 313 patients who meet the inclusion criteria was performed between January 2012 and December 2018. Inclusive criteria: (1) pathologically proven primary NPC; (2) available Immunohistochemistry (IHC) results of EGFR expression situation for primary lesion; (3) radical radiotherapy; (4) whole-body PET/CT before treatment. The exclusion criteria were: (1) received anti-EGFR targeted therapy; (2) received conventional 2D/3D radiotherapy; (3) > 70 years old or < 18 years old; (4) de novo metastatic NPC; (5) with second primary carcinoma or severe medical complications; (6) pregnancy or lactation; (7) disrupted treatment. The flowchart of the study is displayed in Additional file 1: Fig. S1. Patients were re-staged based on the eighth edition TNM staging of the American Joint Committee on Cancer (AJCC). The ethics committee of Fujian Cancer Hospital reviewed and approved the study (No. YKT2020-011-01).

### IHC analysis

All tissue samples were sliced into 4-μm serial sections after fixation in formalin and paraffin embedding [21]. Sections with hematoxylin and eosin staining were used for diagnosis and IHC analysis. All tissue sections were soaked in xylene for deparaffinization before staining. Alcohols with a series of concentration gradients were used for rehydration.Heat-induced epitope retrieval techniques were used for antigen retrieval.The slides were incubated overnight at 4℃ with anti-EGFR monoclonal antibodies (Santa Cruz Biotechnology, Santa Cruz, CA, USA) at a dilution of 1:30.

NPC tissues previously expressing EGFR were used as positive control, and normal serum staining results instead of antibody were used as negative control [21]. Expression levels were estimated by assessing the percentage of tumor cell membrane for EGFR staining. Expression was considered positive if > 10% of cancer cells showed immunoreactivity.

### ^18^F‑FDG PET/CT imaging

All patients underwent ^18^F-FDG PET/CT on a Gemini TF 64 PET/CT scanner (Philips, The Netherlands) [22]. PET images were iteratively reconstructed using CT-based attenuation correction.

The ^18^F-FDG SUV was calculated as [(decay-corrected activity/tissue volume)/(injected dose/body weight)][22]. SUVmax-T and SUVmax-N were defined as the value of the most intense voxel within the region of interest by visually placing the volume of interest.

### Treatment

Radiotherapy dose and target volume delineation were performed according to the recommendations (Radiation Therapy Oncology Group) [22, 23]. The total dose of the planning target volume (PTV) was 69.7–70.0 Gy for primary gross tumor volume or gross tumor volume of lymph nodes, 60–62.7 Gy for the high-risk region (CTV1), and 54.4–56.2 Gy for the low-risk region (CTV2). We divided the total dose into 33–35 fractions.

Patients with stage I were treated with radiotherapy alone, while patients with stage II-IV received radiotherapy combined with chemotherapy [23].

### Follow‑up and clinical endpoints

After RT, follow-up was conducted once every 3 months for the first 2 years, once every 6 months in years 3 to 5, and annually thereafter. The final follow-up date was March 2022. Study endpoints included local recurrence-free survival (LRFS), regional recurrence-free survival (RRFS), locoregional relapse-free survival (LRRFS), distant metastasis-free survival (DMFS), progression-free survival (PFS), and OS.

### Statistical analysis

Uses IBM SPSS statistical software version 22.0 and R software version 4.0.5 for the statistical analysis. The optimal cutoff values was decided by Time-dependent receiver operator characteristic (ROC) analysis (survivalROC package). Kaplan–Meier methods were used to compute the survival analyses. Between-group differences in survival outcomes were assessed using log-rank tests. Cox regression was used to screen out multiple risk factors. All tests were two-tailed, and *P*-values < 0.05 indicated statistical significance.

## Results

### Patients’ characteristics

The clinical characteristics of all eligible patients are summarized in Table 1. This study included 313 patients. After a median follow-up period of 38 months (range, 6–96 months), 24 developed local recurrence, 17 developed regional recurrence, 32 exhibited distant metastases and 26 patients died. The 3-year LRFS, RRFS, LRRFS, DMFS, PFS, and OS rates were 93.2%, 93.7%, 89.2%, 89.6%, 80.1%, and 92.1%, respectively.

**Table 1:** The clinical characteristics of all 313 eligible patients

Characteristic	*N*	%
*Sex*		
Male	230	73.4
Female	83	26.5
*Age(y)*		
Median (range)	50(19–70)	
≥ 50	159	50.7
< 50	154	49.2
*Tumor category*		
T1	63	20.1
T2	67	21.4
T3	119	38.0
T4	64	20.4
*Node category*		
N0	28	8.9
N1	101	32.2
N2	114	36.4
N3	70	22.3
*Clinical stage*		
I	13	4.1
II	34	10.8
III	140	44.7
IVa	126	40.2
*EGFR expression*		
Positive	241	77.0
Negative	72	23.0
*SUVmax-t*		
Mean ± SD	10.21 ± 5.59	
*SUVmax-n*		
Mean ± SD	7.53 ± 5.55	

### IHC result of EGFR expression and survival outcomes

Among 1877 patients with NPC with IHC examination for primary lesions, EGFR was detected in 73.36% (1377 patients), and the proportion of patients showing negligible intensity (negative) of EGFR staining was 26.64% (500 patients). For the enrolled 313 patients, 241 (77.00%) showed positive EGFR expression, while 72 (23.00%) showed negligible expression (negative). The 3-year LRFS, RRFS, LRRFS, DMFS, PFS, and OS rates in the EGFR-negative group vs. EGFR-positive group were 96.9% vs. 92.2% (*P* = 0.075), 97.9% vs. 93.2% (*P* = 0.084), 94.7% vs. 87.6% (*P* = 0.032), 97.0% vs. 87.4% (*P* = 0.018), 91.8% vs. 76.7% (*P* = 0.0017), and 96.5% vs. 90.8% (*P* = 0.058), respectively (Additional file 1: Fig. S2). The LRRFS, DMFS, and PFS in the EGFR-negative group tend to be better than those in the EGFR-positive group (*P* < 0.05).

### Time-dependent ROC analysis determines the best cutoff value of SUVmax

**Fig. 1:**
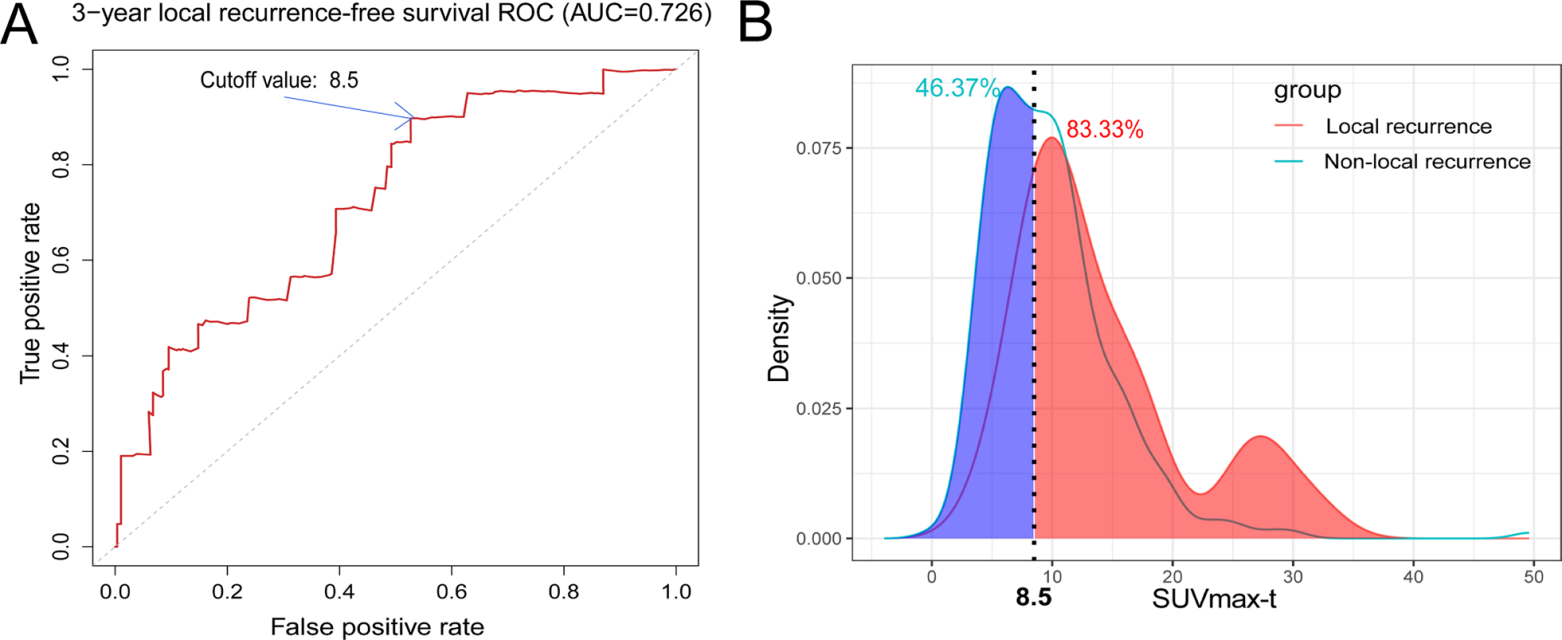
The optimal cutoff value of SUVmax-T. **A** The optimal cutoff value of SUVmax-T for predicting 3-year local recurrence-free survival (LRFS) was 8.5; **B** Probability density functions with an SUVmax-T threshold of 8.5 for predicting local recurrence

The mean SUVmax-T was 10.21 ± 5.59 (range, 2.50–49.6), and the mean SUVmax-N was 7.53 ± 5.55 (range, 0–36.6). To further evaluate the prognostic value of SUVmax, time-dependent ROC analysis was used to determine the optimal cutoff values based on the 3-year survival outcome. The optimal cutoff value of SUVmax-T was 8.5 based on the 3-year LRFS (area under the curve = 0.726, Fig. 1A). When SUVmax-T threshold was set at 8.5, we could accurately screen about 83.33% of local recurrence patients and 46.37% of patients without local recurrence risk (Fig. 1B). The 3-year LRFS, RRFS, LRRFS, DMFS, PFS, and OS rates for the low SUVmax-T (≤ 8.5) group vs. the high SUVmax-T (> 8.5) group were 96.7% and 89.1% (*p* = 0.0038), 96.3% vs. 95.3% (*p* = 0.21), 93.8% vs. 84.2% (*p* = 0.0063), 93.5% vs. 86.5% (*p* = 0.022), 85.8% vs. 75.5% (*p* = 0.018), and 94.4% vs. 93.5 (*p* = 0.066), respectively (Additional file 1: Fig. S3). LRFS, LRRFS, DMFS, and PFS in the low SUVmax-T group were significantly better than those in the high SUVmax-T group (*p* < 0.05).

### Prognostic value of SUVmax-T combined with EGFR expression

**Fig. 2:**
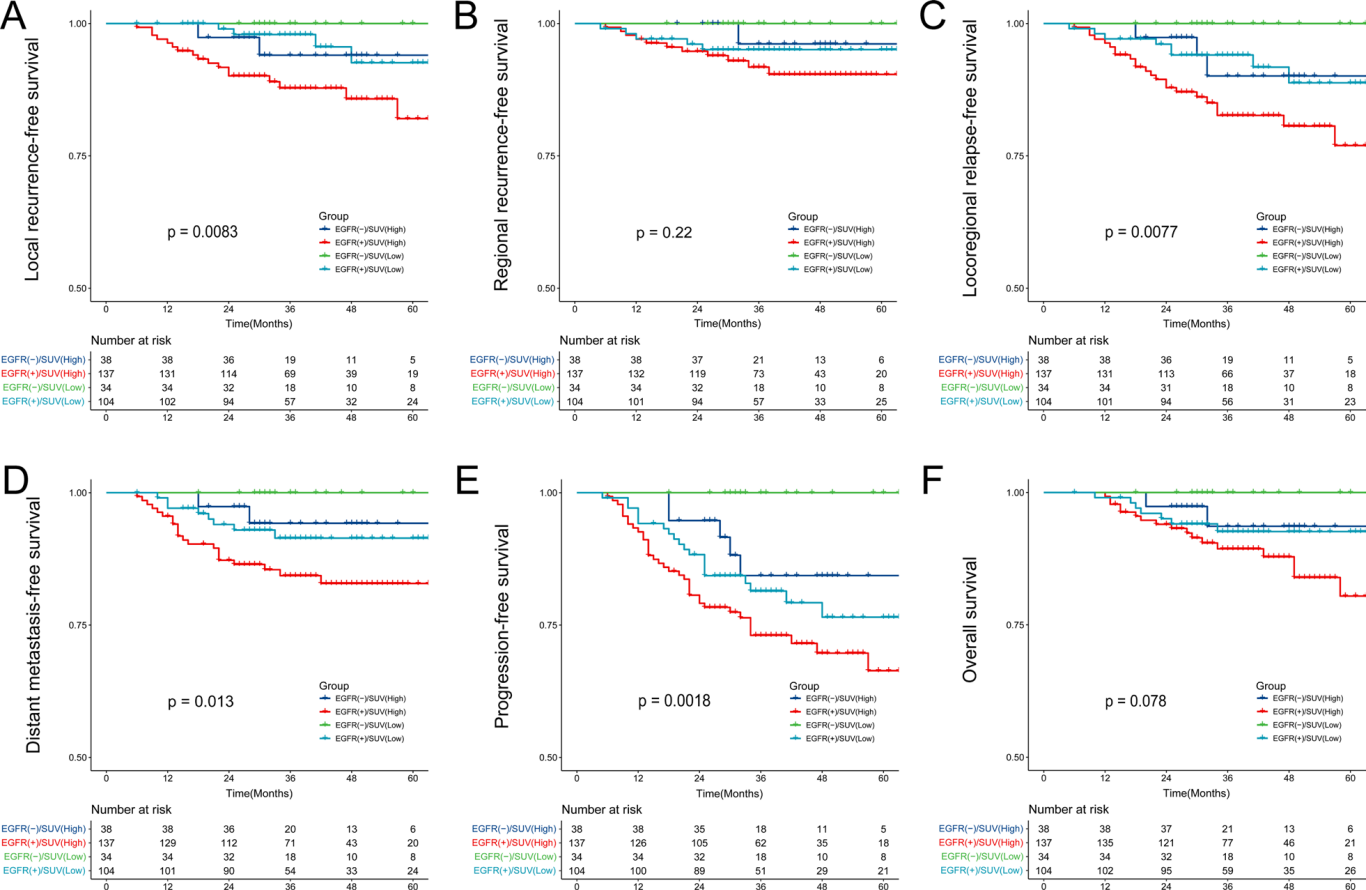
Kaplan–Meier curves among the four groups according to SUVmax-T (≤ 8.5 or > 8.5) and EGFR expression (negative or positive). **A** local recurrence-free survival (LRFS); **B** regional recurrence-free survival (RRFS); **C** locoregional relapse-free survival (LRRFS); **D** distant metastasis-free survival (DMFS); **E** progression-free survival (PFS); (F) overall survival (OS)

To better predict the prognosis of NPC, patients were divided into the following four groups based on SUVmax-T and EGFR expression: (a) EGFR negative and low SUVmax-T, (b) EGFR negative and high SUVmax-T, (c) EGFR positive and low SUVmax-T, and (d) EGFR positive and high SUVmax-T. There were obviously statistical difference in 3-year LRFS (*p* = 0.0083), LRRFS (*p* = 0.0077), DMFS (*p* = 0.013), and PFS (*p* = 0.0018) (Fig. 2) among the four groups. The 3-year PFS rates for these four groups were as follows: (a) 100%, (b) 86.8%, (c) 82.7%, and (d) 75.2% (*p* = 0.0018).

**Fig. 3:**
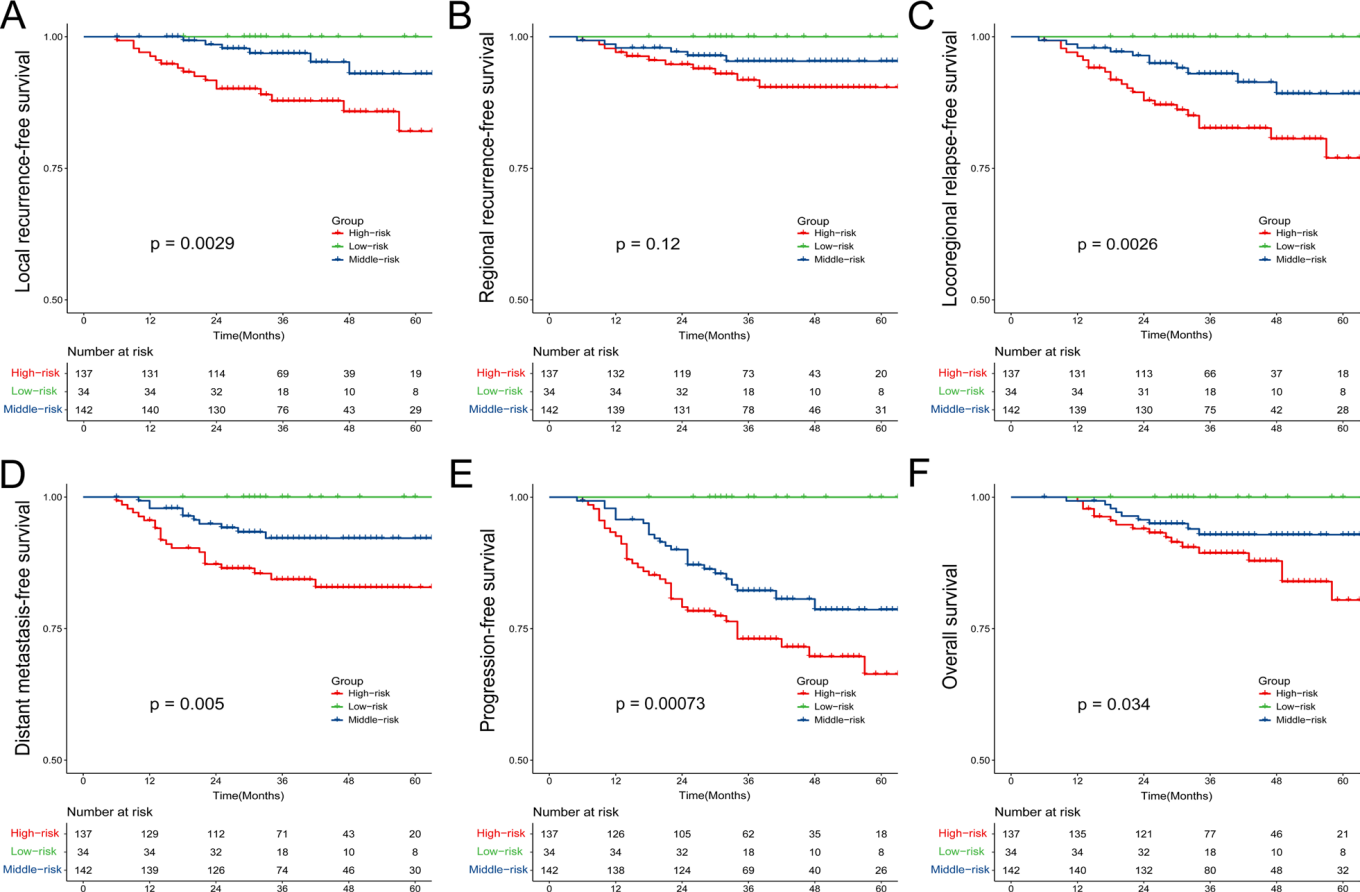
Kaplan–Meier curves among 3 risk group. **A** local recurrence-free survival (LRFS); **B** regional recurrence-free survival (RRFS); **C** locoregional relapse-free survival (LRRFS); **D** distant metastasis-free survival (DMFS); **E** progression-free survival (PFS); **F** overall survival (OS)

Figure 2 shows that the survival curves for the (b) EGFR-negative and high SUVmax-T and (c) EGFR-positive and low SUVmax-T were extremely close, while these two curves diverge conspicuously from the survival curve of (a) EGFR-negative and low SUVmax-T and (d) EGFR-positive and high SUVmax-T. Then all patients were divided into three groups: patients in (a) group were defined as the low-risk group, patients in (b) and (c) groups were defined as the middle-risk group, and patients in (d) group were defined as the low-risk group. In addition to the 3-year LRFS (*p* = 0.0029), LRRFS (*p* = 0.0026), DMFS (*p* = 0.005), and PFS (*p* = 0.00073), the 3-year OS also showed significant difference among 3 risk groups (*p* = 0.034, Fig. 3). The 3-year OS rate was 100% in low-risk group, 93.7% in medium-risk group and 89.8% in high-risk group, respectively (*p* = 0.034).

### Univariate and multivariate analysis

Six variables (age, T-stage, N-stage, EGFR expression, SUVmax-T, and SUVmax-N) were included in the univariate analysis for the six clinical endpoints, and the results are summarized in Additional file 1: Table 1. Variables that were significantly associated with each endpoint were included in the multivariate analysis (Table 2). Multivariate analysis indicated that SUVmax-T was an independent prognostic factor for LRFS (hazard ratio [HR] = 1.060; 95% confidence interval [CI],1.018–1.103; *p* = 0.005) and LRRFS (HR = 1.076; 95% CI, 1.010–1.147; *P* = 0.023). Moreover, multivariable survival analysis revealed that EGFR was an independent prognostic factor for LRRFS (HR = 3.409; 95% CI 1.019–11.402; *P* = 0.046), DMFS (HR = 4.497; 95% CI 1.067–18.960; *P* = 0.041), and PFS (HR = 3.851; 95% CI 1.524–9.730; *P* = 0.004).

**Table 2:** Multivariate analysis of LRFS, RRFS, LRRFS, DMFS, PFS and OS

Variables		Multivariate analysis	
		*P*	HR (95%CI)
*Test for LRFS*			
T-stage	T1-T2 vs. T3-T4	0.055	2.975(0.974–9.084)
EGFR	Negative vs. positive	0.147	1.074(0.684–12.536)
SUVmax-T	–	0.005	1.060(1.018–1.103)
*Test for RRFS*			
N-stage	N0-N1 vs. N2-N3	0.023	12.950(1.423–111.833)
SUVmax-T	–	0.526	1.028(0.943–1.120)
SUVmax-N	–	0.025	1.110(1.013–1.216)
*Test for LRRFS*			
T-stage	T1-T2 vs. T3-T4	0.503	1.310(0.594–2.892)
N-stage	N0-N1 vs. N2-N3	0.007	3.392(1.389–8.280)
EGFR	Negative vs. positive	0.046	3.409(1.019–11.402)
SUVmax-T	–	0.023	1.076(1.010–1.147)
SUVmax-N	–	0.901	1.004(0.937–1.077)
*Test for DMFS*			
T-stage	T1-T2 vs. T3-T4	0.045	2.303(1.020–5.198)
N-stage	N0-N1 vs. N2-N3	0.002	24.741(3.349–182.792)
EGFR	Negative vs. positive	0.041	4.497(1.067–18.960)
SUVmax-N	–	0.521	1.021(0.957–1.090)
*Test for PFS*			
T-stage	T1-T2 vs. T3-T4	0.113	1.603(0.895–2.871)
N-stage	N0-N1 vs. N2-N3	< 0.001	4.060(2.060–8.004)
EGFR	Negative vs. positive	0.004	3.851(1.524–9.730)
SUVmax-T	–	0.073	1.046(0.996–1.099)
SUVmax-N	–	0.588	1.014(0.964–1.067)
*Test for OS*			
Age	< 50 vs. ≥ 50	0.034	2.484(1.070–5.768)
T-stage	T1-T2 vs. T3-T4	0.229	1.817(0.687–4.806)
N-stage	N0-N1 vs. N2-N3	0.009	4.513(1.460–13.951)
EGFR	Negative vs. positive	0.099	3.446(0.793–14.972)
SUVmax-T	–	0.361	1.036(0.960–1.119)
SUVmax-N	–	0.227	1.049(0.971–1.133)

*SUVmax-T* Standardized uptake value of the primary tumor, *SUV max-N* The highest standardized uptake value of neck lymph nodes, *DMFS* Distant metastasis-free survival, *LRFS* Local recurrence‐free, survival; *RRFS* Regional recurrence-free survival, *LRRFS* Locoregional relapse-free survival, *DMFS* Distant metastasis-free survival, *PFS* Progression-free survival, *OS* Overall survival

## Discussion

The most important prognostic factor for NPC is TNM clinical stage. However, there is considerable variability in outcomes among patients with the same TNM stage receiving the same treatment [24]. Recent studies have indicated that other prognostic factors, such as plasma Epstein-Barr virus-DNA copy numbers and PET/CT parameters, may be used as supplementary index to fix the current staging system [25–27]. Few studies have combined functional imaging data with molecular pathological data to predict NPC prognosis. This is the first study to combine EGFR expression of the primary lesion and pretreatment SUVmax of PET/CT to predict prognosis and successfully proved that it is a potential predictor.

PET/CT is functional imaging, which is different from morphology and structure imaging. The parameters of PET can be used to characterize the burden of metabolically active lesions and biological aggressiveness in malignancies. SUV, TLG, and MTV are parameters that have been correlated with survival outcome [12–16]. SUVmax has the advantages of measure convenient, consistency and repeatability. It is the most representative parameter and reflects the highest metabolic activity. Recently, other volume-based parameters such as TLG, SUVpeak and MTV have also been shown to be associated with prognosis [28–31]. For instance, Chan et al. confirmed that TLG is an independent prognostic factor for OS in NPC [31]. However, these volume parameters have not been consistently recognized, and some studies have shown that MTV and TLG have no significant predictive value for the prognosis of NPC [32–34]. Thus, we selected SUVmax as the main variable in this study.

In 2008, a retrospective study have shown SUVmax may predict DFS in NPC treated with CCRT and more aggressive treatment should be given to patients with higher SUVmax [35]. A prospective study reported that combination of SUVmax and tumor stage can more accurately predict the treatment outcome in NPC [36]. Consistent with previous published studies, the present study indicated that SUVmax can reflects tumor aggressiveness and has prognostic value in NPC. Different from previous research, recurrence was used as the endpoint of tumor invasiveness to determine the optimal cutoff for SUVmax. Particularly, 3-year LRFS was used to select the best cutoff value for SUVmax-T using time-dependent ROC analysis. In our study, 313 enrolled patients were divided into two groups depending on the optimal cutoff of SUVmax-T. The survival analysis showed that NPC patients with a higher SUVmax-T (> 8.5) had worse LRFS, LRRFS, DMFS, and PFS. This suggests that primary tumors with higher FDG uptake can be more aggressive; therefore, patients may need more intensive treatment and follow-up.

Several meta-analyses have shown that EGFR overexpression is significantly associated with poor OS and DFS. Thus, EGFR may serve as a potential prognostic predictor of NPC [17, 37, 38]. A phase 1 nonrandomized clinical trial suggested that MRG003 (an anti-EGFR antibody) showed promising antitumor activity in patients with EGFR-positive NPC [39]. Other monoclonal antibody drugs targeting EGFR, such as nimotuzumab and cetuximab, also showed satisfactory clinical benefits and a manageable safety profile for advanced or recurrent/metastatic NPC [40, 41]. To eliminate the effect of anti-EGFR antibodies on prognosis, we excluded patients who received anti-EGFR targeted therapy. Ultimately, survival analysis demonstrated that EGFR-positive expression in primary lesions was significantly related to poor treatment outcomes and higher aggressiveness.

To better predict the clinical outcomes of de novo NPC, we combined functional imaging data with molecular pathological data. Patients were categorized into four groups based on EGFR expression (negative or positive) and SUVmax-T (≤ 8.5 or > 8.5). There were significant differences in 3-year LRFS (*p* = 0.0083), LRRFS (*p* = 0.0077), DMFS (*p* = 0.013), and PFS (*p* = 0.0018) among the four groups. The K–M curve revealed that patients in the EGFR-positive and SUVmax-T > 8.5 group had the worst survival, while patients in the EGFR-negative and SUVmax-T ≤ 8.5 group had the best prognosis. For patients in the EGFR-negative + SUVmax-T > 8.5 group and EGFR-positive + SUVmax-T ≤ 8.5 group, the cumulative survival curves were extremely close. This demonstrated that EGFR expression and high SUVmax-T may be adverse prognostic factors for NPC. Therefore, we further divided patients into three groups. Patients with positive EGFR expression or high SUVmax-T levels were defined as the middle-risk group. The 3-year OS also showed a significant difference among the three risk groups (*p* = 0.034). Moreover, SUVmax-T was associated with LRFS and LRRFS in multivariate analysis, whereas EGFR was an independent prognostic factor for LRRFS, DMFS, and PFS.

High SUVmax-T and EGFR expression of the primary tumor were correlated with highly invasive and poor clinical outcomes, especially LRRFS. Chan et al. proved that the combined information of SUVmax and tumor staging can guide the use of neoadjuvant/adjuvant therapy and surveillance protocols to improve distant control [42]. A large cohort retrospective analysis suggested that the addition of anti-EGFR targeted treatment to CCRT is more effective for maximizing survival in NPC patients compared with CCRT alone [43]. Xia et al. also revealed that a significant DMFS benefit with CCRT plus anti-EGFR targeted in patients with N2-N3 stage [44]. Therefore, a more aggressive systematic treatment for patients with EGFR expression and high SUVmax-T is warranted. However, further prospective investigations are required to determine whether combined information of EGFR and SUVmax can guide the use of anti-EGFR targeted treatment or ncreasing the radiation dose to improve survival outcomes.

The advantages of this study include the invasive combination of EGFR expression and pretreatment with PET/CT and a relatively large sample size. The current study did have some limitations. First, as this was a retrospective study, our results may have been affected by selection bias. Second, the cutoff value of SUV may vary from institution to institution depending on different PET/CT scanners and protocols. Lastly, the current study enrolled a relatively low number of EGFR-negative patients, owing to EGFR overexpression in NPC.

## Conclusion

High SUVmax-T and EGFR expression in primary lesions is associated with significantly worse survival in NPC. The combination of SUVmax-T and EGFR expression can refine prognosis and indicate clinical therapy. This finding might lead to an improved risk stratification and identify patients who require individual treatment to reduce the risk of treatment failure.

## Supplementary Information

**Additional file 1: Table S1** Univariate analysis of LRFS, RRFS, LRRFS, DMFS, PFS and OS. **Fig. S1**: The flowchart of the present study. **Fig. S2**: Kaplan-Meier curves in the EGFR-negative group and the EGFR-positive group. **A** local recurrence-free survival (LRFS); **B** regional recurrence-free survival (RRFS); **C **locoregional relapse-free survival (LRRFS); **D** distant metastasis-free survival (DMFS); **E** progression-free survival (PFS); **F** overall survival (OS). **Fig. S3**: Kaplan-Meier curves in the low SUVmax-T (≤8.5) group and the high SUVmax-T (>8.5) group. **A** local recurrence-free survival (LRFS); **B** regional recurrence-free survival (RRFS); **C** locoregional relapse-free survival (LRRFS); **D** distant metastasis-free survival (DMFS); **E** progression-free survival (PFS); **F** overall survival (OS).

## Data Availability

Data are available upon reasonable request. The data sets generated during and/or analyzed during the current study are available from the corresponding author on reasonable request.

## Abbreviations

NPC: Nasopharyngeal carcinoma
CCRT: Concurrent chemoradiotherapy
RT: Radiotherapy
IMRT: Intensity-modulated RT
OS: Overall survival
^18^F-FDG PET/CT: ^18^F-fluorodeoxyglucose positron emission tomography and computed tomography
SUV: Standardized uptake value
TLG: Termed total lesion glycolysis
MTV: Metabolic tumor volume
SUVmax: Maximum standard uptake value
EGFR: Epidermal growth factor receptor
IHC: Immunohistochemical
AJCC: American joint committee on cancer
PTV: Planning target volume
NAC: Neoadjuvant chemotherapy
LRFS: Local recurrence-free survival
RRFS: Regional recurrence-free survival
LRRFS: Locoregional relapse-free survival
DMFS: Distant metastasis-free survival
PFS: Progression-free survival
ROC: Receiver operator characteristic
K-M: Kaplan–Meier
DFS: Disease-free survival
