# Supplementary material for: PET/CT standardized uptake value and EGFR expression predicts treatment failure in nasopharyngeal carcinoma
Source: Radiat Oncol. 2023 Feb 22;18:33. doi: 10.1186/s13014-023-02231-6 (PMC9945369; doi:10.1186/s13014-023-02231-6)

**Supplementary Table 1.** Univariate analysis of LRFS, RRFS, LRRFS, DMFS, PFS and OS.

| **Variables** |  | **Univariate analysis** | | |
| --- | --- | --- | --- | --- |
|  |  |  | P | HR (95%CI) |
| **Test for LRFS** |  |  |  |  |
| Age | <50 vs. ≥50 |  | 0.634 | 0.823(0.368-1.837) |
| T-stage | T1-T2 vs. T3-T4 |  | 0.010 | 4.179(1.413-12.358) |
| N-stage | N0-N1 vs. N2-N3 |  | 0.516 | 1.316(0.575-3.013) |
| EGFR | Negative vs. positive |  | 0.095 | 3.434(0.807-14.604) |
| SUVmax-T | - |  | <0.001 | 1.075(1.035-1.116) |
| SUVmax-N | - |  | 0.507 | 1.023(0.956-1.094) |
| **Test for RRFS** |  |  |  |  |
| Age | <50 vs. ≥50 |  | 0.777 | 0.871(0.336-2.259) |
| T-stage | T1-T2 vs. T3-T4 |  | 0.903 | 1.062(0.404-2.791) |
| N-stage | N0-N1 vs. N2-N3 |  | 0.016 | 11.912(1.580-89.830) |
| EGFR | Negative vs. positive |  | 0.120 | 4.968(0.659-37.469) |
| SUVmax-T | - |  | 0.018 | 1.063(1.011-1.118) |
| SUVmax-N | - |  | <0.001 | 1.116(1.061-1.175) |
| **Test for LRRFS** |  |  |  |  |
| Age | <50 vs. ≥50 |  | 0.917 | 1.036(0.534-2.010) |
| T-stage | T1-T2 vs. T3-T4 |  | 0.067 | 1.994(0.953-4.174) |
| N-stage | N0-N1 vs. N2-N3 |  | 0.032 | 2.294(1.074-4.902) |
| EGFR | Negative vs. positive |  | 0.044 | 3.369(1.032-11.003) |
| SUVmax-T | - |  | <0.001 | 1.064(1.028-1.102) |
| SUVmax-N | - |  | 0.001 | 1.075(1.029-1.123) |
| **Test for DMFS** |  |  |  |  |
| Age | <50 vs. ≥50 |  | 0.144 | 0.587(0.287-1.200) |
| T-stage | T1-T2 vs. T3-T4 |  | 0.040 | 2.320(1.040-5.176) |
| N-stage | N0-N1 vs. N2-N3 |  | 0.002 | 24.418(3.333-178.902) |
| EGFR | Negative vs. positive |  | 0.032 | 4.797(1.146-20.076) |
| SUVmax-T | - |  | 0.250 | 1.030(0.979-1.084) |
| SUVmax-N | - |  | 0.023 | 1.058(1.008-1.112) |
| **Test for PFS** |  |  |  |  |
| Age | <50 vs. ≥50 |  | 0.933 | 0.979(0.600-1.599) |
| T-stage | T1-T2 vs. T3-T4 |  | 0.009 | 2.068(1.195-3.579) |
| N-stage | N0-N1 vs. N2-N3 |  | <0.001 | 3.257(1.770-5.992) |
| EGFR | Negative vs. positive |  | 0.004 | 3.867(1.552-9.636) |
| SUVmax-T | - |  | 0.008 | 1.044(1.011-1.077) |
| SUVmax-N | - |  | 0.001 | 1.062(1.027-1.099) |
| **Test for OS** |  |  |  |  |
| Age | <50 vs. ≥50 |  | 0.059 | 2.233(0.971-5.137) |
| T-stage | T1-T2 vs. T3-T4 |  | 0.047 | 2.523(1.012-6.286) |
| N-stage | N0-N1 vs. N2-N3 |  | 0.008 | 4.220(1.453-12.257) |
| EGFR | Negative vs. positive |  | 0.077 | 3.680(0.870-15.577) |
| SUVmax-T | - |  | 0.028 | 1.052(1.006-1.100) |
| SUVmax-N | - |  | 0.001 | 1.084(1.033-1.136) |

**Abbreviations:** SUVmax-T, standardized uptake value of the primary tumor; SUV max-N, the highest standardized uptake value of neck lymph nodes; DMFS, distant metastasis-free survival; LRFS, local recurrence‐free survival; RRFS, regional recurrence-free survival; LRRFS, locoregional relapse-free survival; DMFS, distant metastasis-free survival; PFS, progression-free survival; OS, overall survival.

**Supplementary Figure Captions:**

**Supplementary Fig.1**: The flowchart of the present study.

**
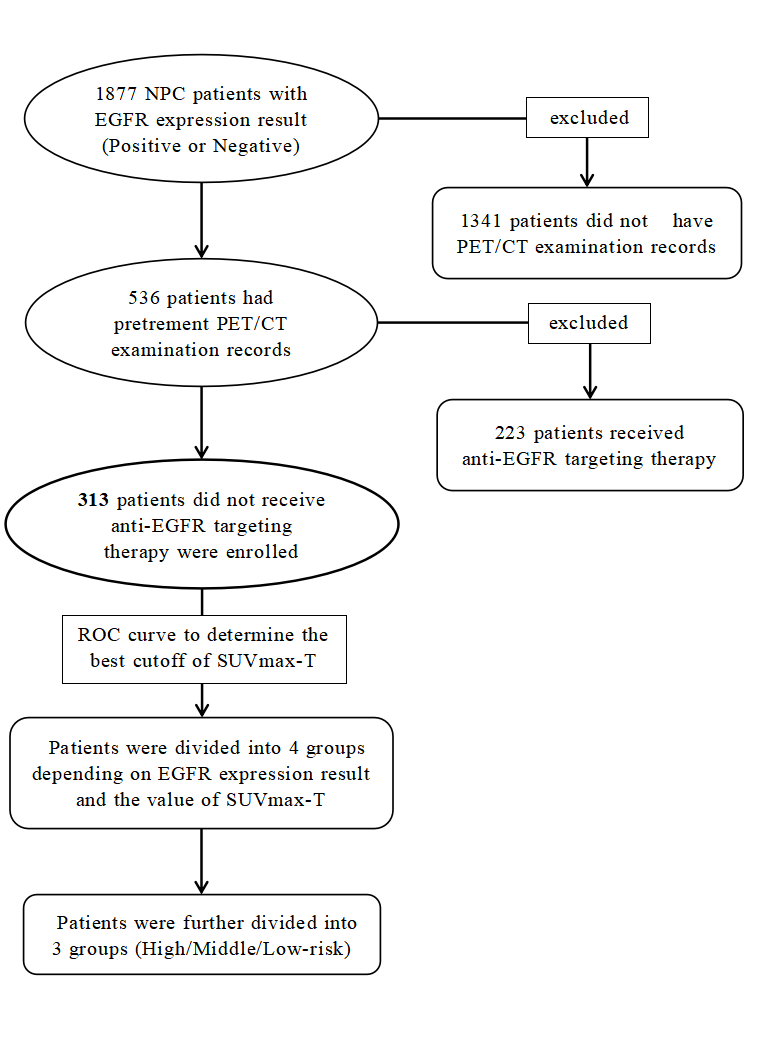
**

**Supplementary Fig.2**: Kaplan-Meier curves in the EGFR-negative group and the EGFR-positive group. (A) local recurrence-free survival (LRFS); (B) regional recurrence-free survival (RRFS); (C)locoregional relapse-free survival (LRRFS); (D) distant metastasis-free survival (DMFS); (E) progression-free survival (PFS); (F) overall survival (OS).


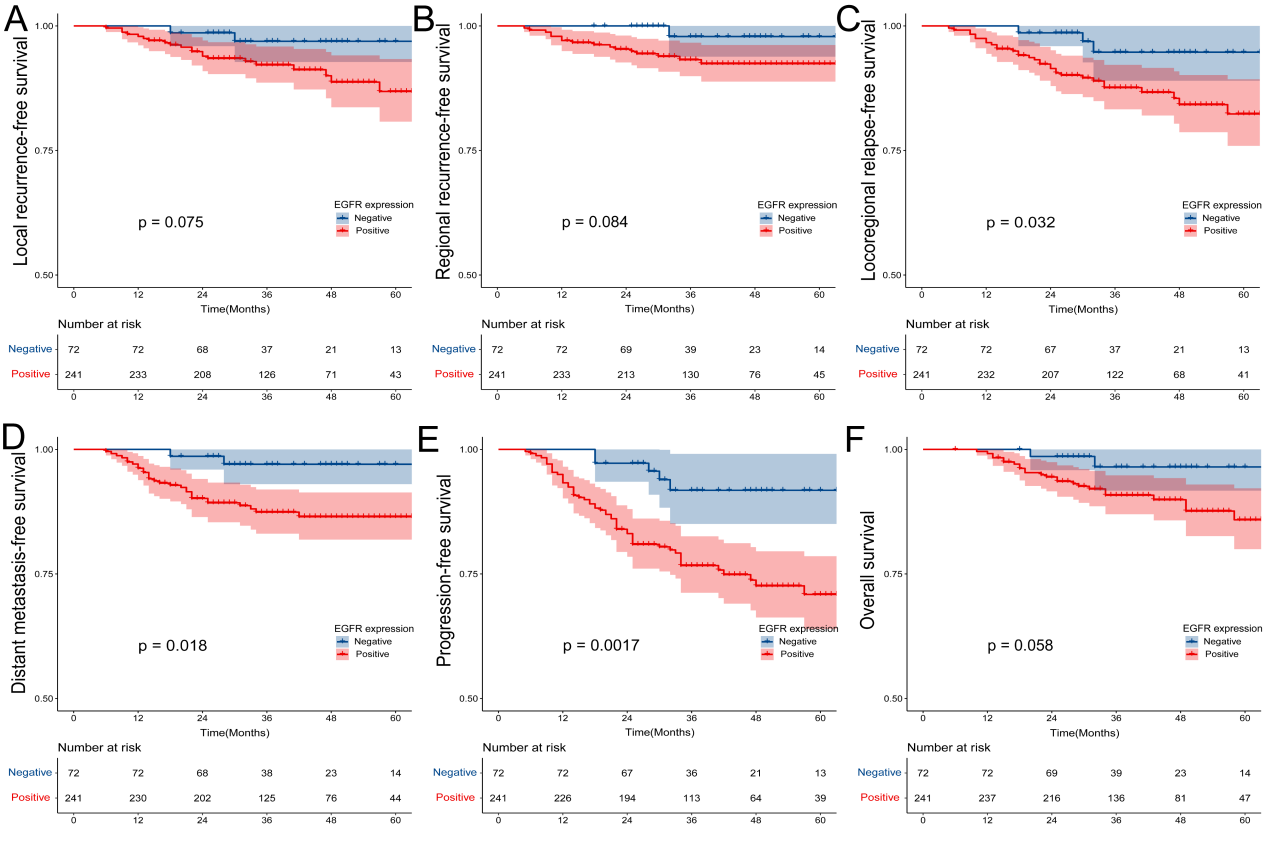


**Supplementary Fig.3**: Kaplan-Meier curves in the low SUVmax-T (≤8.5) group and the high SUVmax-T (>8.5) group . (A) local recurrence-free survival (LRFS); (B) regional recurrence-free survival (RRFS); (C)locoregional relapse-free survival (LRRFS); (D) distant metastasis-free survival (DMFS); (E) progression-free survival (PFS); (F) overall survival (OS).


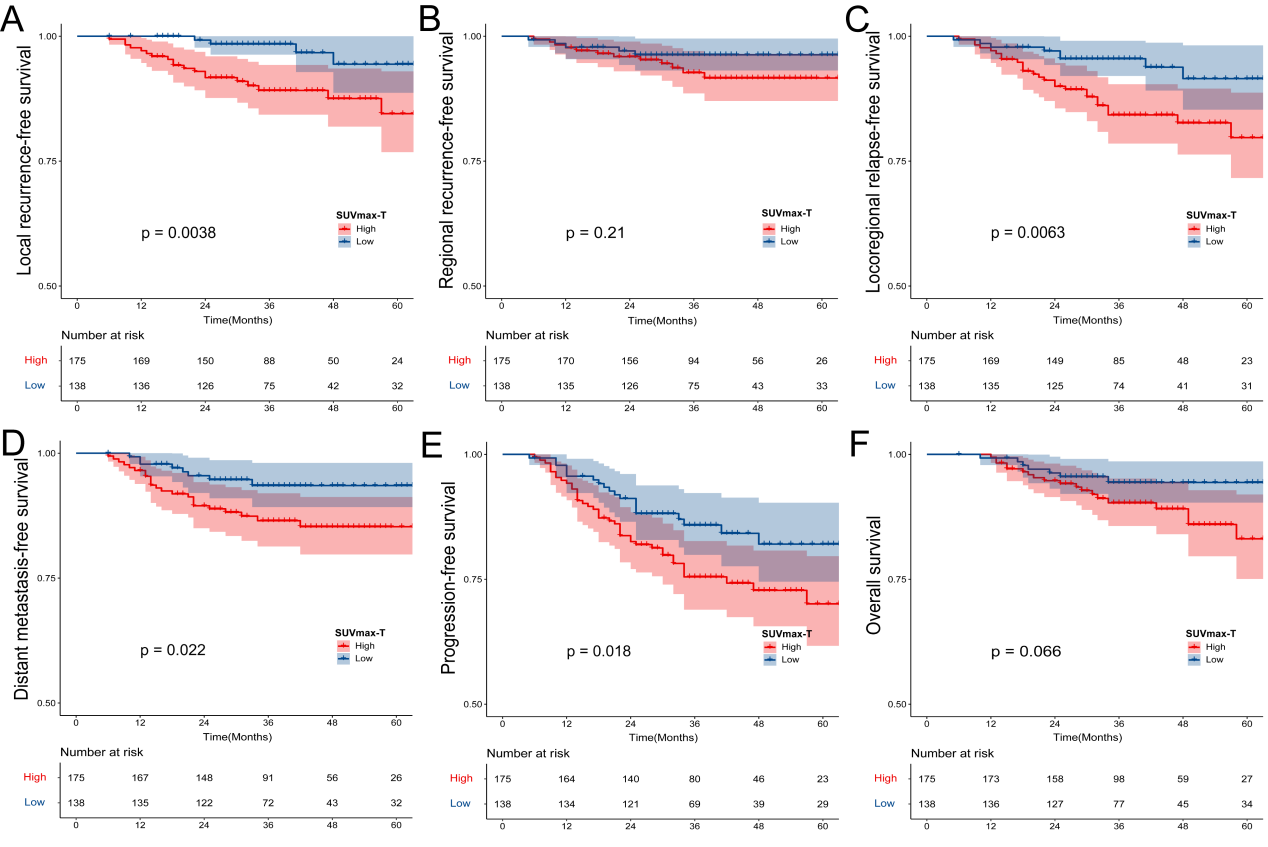

Supplement: Supplementary file 1 — Additional file 1: Table S1 Univariate analysis of LRFS, RRFS, LRRFS, DMFS, PFS and OS. Fig. S1: The flowchart of the present study. Fig. S2: Kaplan-Meier curves in the EGFR-negative group and the EGFR-positive group. A local recurrence-free survival (LRFS); B regional recurrence-free survival (RRFS); C locoregional relapse-free survival (LRRFS); D distant metastasis-free survival (DMFS); E progression-free survival (PFS); F overall survival (OS). Fig. S3: Kaplan-Meier curves in the low SUVmax-T (≤8.5) group and the high SUVmax-T (>8.5) group. A local recurrence-free survival (LRFS); B regional recurrence-free survival (RRFS); C locoregional relapse-free survival (LRRFS); D distant metastasis-free survival (DMFS); E progression-free survival (PFS); F overall survival (OS). [file 13014_2023_2231_MOESM1_ESM.docx]
